# Contrast-free optical coherence tomography:Systematic evaluation of non-contrast media for intravascular assessment

**DOI:** 10.1371/journal.pone.0237588

**Published:** 2020-08-20

**Authors:** Trevor Simard, Pouya Motazedian, Kamran Majeed, Kiran Sarathy, Richard G. Jung, Joshua Feder, F. Daniel Ramirez, Pietro Di Santo, Jeffrey Marbach, Shan Dhaliwal, Spencer Short, Alisha Labinaz, Carl Schultz, Juan J. Russo, Derek So, Aun-Yeong Chong, Michel Le May, Benjamin Hibbert

**Affiliations:** 1 CAPITAL Research Group, Division of Cardiology, University of Ottawa Heart Institute, Ottawa, Ontario, Canada; 2 Department of Cellular and Molecular Medicine, University of Ottawa, Ottawa, Ontario, Canada; 3 Cumming School of Medicine, University of Calgary, Calgary, Alberta, Canada; 4 Department of Cardiology, Royal Perth Hospital, Perth, Western Australia, Australia; 5 School of Medicine, University of Western Australia, Perth, Western Australia, Australia; 6 Hôpital Cardiologique du Haut-Lévêque, CHU Bordeaux, Bordeaux-Pessac, France; 7 L’Institut de Rythmologie et Modélisation Cardiaque (LIRYC), Université de Bordeaux, Bordeaux-Pessac, France; Nicolaus Copernicus University, POLAND

## Abstract

**Background:**

Coronary revascularization using imaging guidance is rapidly becoming the standard of care. Intravascular optical coherence tomography uses near-infrared light to obtain high resolution intravascular images. Standard optical coherence tomography imaging technique employs iodinated contrast dye to achieve the required blood clearance during acquisition. We sought to systematically evaluate the technical performance of saline as an alternative to iodinated contrast for intravascular optical coherence tomography assessment.

**Methods and results:**

We performed bench top optical coherence tomography analysis on nylon tubing with sequential contrast/saline dilutions to empirically derive adjustment coefficients. We then applied these coefficients *in vivo* in an established rabbit abdominal stenting model with both saline and contrast optical coherence tomography imaging. In this model, we assessed the impact of saline on both quantitative and qualitative vessel assessment. Nylon tubing assessment demonstrated a linear relationship between saline and contrast for both area and diameter. We then derived adjustment coefficients, allowing for accurate calculation of area and diameter when converting saline into both contrast and reference dimensions. *In vivo* studies confirmed reduced area with saline versus contrast [7.43 (5.67–8.36) mm^2^ versus 8.2 (6.34–9.39) mm^2^, p = 0.001] and diameter [3.08 mm versus 3.23 mm, p = 0.001]. Following correction, a strong relationship was achieved *in vivo* between saline and contrast in both area and diameter without compromising image quality, artefact, or strut assessment.

**Conclusion:**

Saline generates reduced dimensions compared to contrast during intravascular optical coherence tomography imaging. The relationship across physiologic coronary diameters is linear and can be corrected with high fidelity. Saline does not adversely impact image quality, artefact, or strut assessment.

## Introduction

Coronary angiography and percutaneous coronary intervention (PCI) are predominantly performed based on fluoroscopic and cineangiographic images for determining vessel pathology and sizing. Coronary assessment has greatly improved with the advent of intravascular imaging modalities–specifically intravascular ultrasound (IVUS) and intravascular optical coherence tomography (OCT). OCT uses near-infrared light (wavelength ~1300nm) to generate tomographic images with histological-grade resolution (10-20um). This enhanced resolution enables OCT to provide detailed intravascular assessment for thrombus, plaque morphology, intimal lesions (neointima, dissection) and stent evaluation (apposition, sizing, coverage) [1]. However, OCT requires exclusion of intraluminal blood as it causes significant attenuation of light energy owing to absorption by hemoglobin and scattering by red blood cells [2]. Hence, the use of a flushing solution to clear intraluminal blood is needed for intravascular OCT.

Contemporary FD-OCT (frequency or Fourier-domain OCT) provides pullback speeds up to 75mm/sec, enabling scanning of up to 5-7cm of an epicardial coronary vessels depending on the resolution. The first in-human studies of this technology demonstrated that this improved speed eliminates the need for proximal balloon occlusion as a single, high rate bolus injection was sufficient to exclude blood for imaging [3]. During initial development, saline was employed as a flushing medium with subsequent work demonstrating improved blood exclusion and longer imaging durations (~10 seconds) with viscous contrast media over crystalloids [3–5]. As such, viscous contrast media became the standard imaging medium used in commercial FD-OCT protocols.

Optimizing the imaging medium has garnered significant interest recently as a means of improving image quality while minimizing adverse effects. Indeed, the administration of viscous contrast media is not without risk, namely that of acute kidney injury and contrast-induced nephropathy which, though poorly understood, have been linked to long-term adverse events [6]. The side effect profile is dose-dependent, limiting the number and duration of OCT imaging runs that can be performed. This is particularly relevant in complex cases where a significant contrast load may already have been used for angiography, thus limiting OCT assessment in whom it may be the most beneficial. Accordingly, recent efforts have sought to identify alternative imaging mediums with improved side effect profiles, in particular colloids and crystalloids [7, 8]. Herein, we report the systematic evaluation of saline and saline-diluted contrast as an alternative to iodinated-contrast imaging medium for OCT-based vascular imaging.

## Materials and methods

### OCT and imaging media

OCT scans were acquired using a Dragonfly Imaging Catheter (St. Jude Medical, Minnesota, USA) in conjunction with the ILLUMIEN system (St. Jude Medical, Minnesota, UA). Omnipaque 300 mg/mL (GE Healthcare, NJ, USA) was used as contrast media and 0.9% sodium chloride was used as the saline solution with varying dilutions generated via mixtures of these two agents.

### Bench top model for dimensional analysis

**Fig 1:**
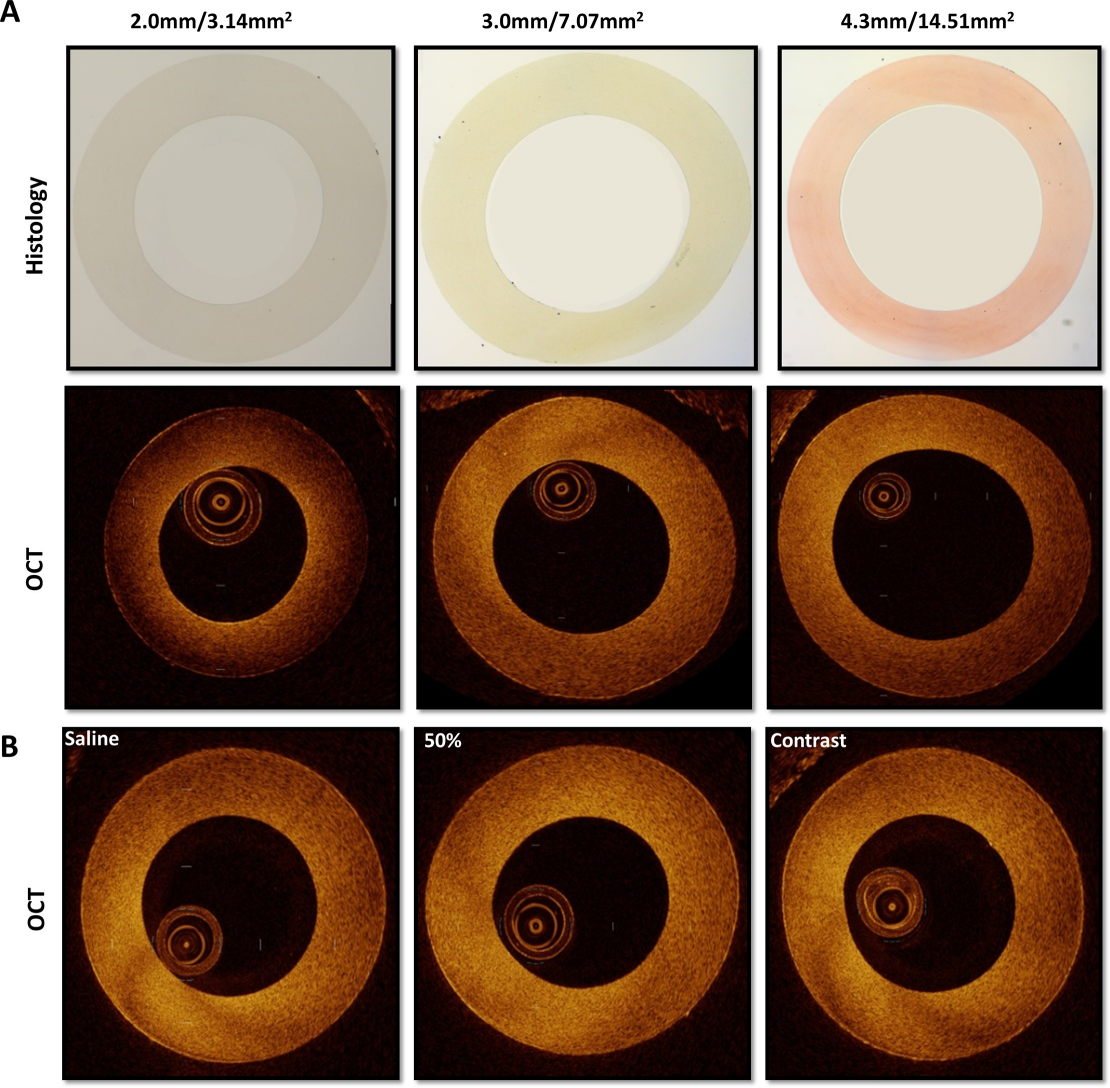
OCT and histological sections of nylon tubing. (A) Representative images of histological cross-sectional images of nylon tubing across physiologic size range for human coronary artery (2.0mm, 3.0mm, and 4.3mm) with corresponding OCT images of these same nylon tubes with 100% contrast flushing agent below. (B) Representative images from a select subset utilizing 100% saline, 50%/50% mixture of saline/contrast, and 100% contrast on identical nylon tubing segment.

Nylon 11 D.O.T. tubing (Freelin-Wade, Oregon, USA) with known internal diameters of 2.0/0.079, 3.0/0.118 and 4.3/0.170 mm/inches (+/- 0.08mm/0.003”) were used to identify the measured differences between saline and contrast injections. We demonstrate histological and OCT cross-sections of each (Fig 1A). Varying solutions were instilled through the tubing for OCT assessment, including 100% saline, varying dilutions of saline/contrast (25%/75%, 50%/50% and 75%/25%) and 100% contrast. Representative images for a selected subset of 100% saline, 50%/50% saline/contrast and 100% contrast demonstrated in Fig 1B. For each tube size and saline/contrast combination, 15 matched images were identified and dimensional analyses were performed to assess the internal area and diameter. This process was performed for both the derivation (n = 15) and validation (n = 15) groups to generate and validate a correction factor between saline and contrast for area and diameter.

### Animals and experimental protocol

All animal protocols are in compliance with the Guide for the Care and Use of Laboratory Animals published by the US National Institutes of Health and the guidelines of the Canadian Council on Animal Care. The animal protocols were approved by the University of Ottawa Animal Care Committee. Rabbits are known to have abdominal aortas and iliac vessels similar in sizing to human coronaries [9, 10], making them a viable model for studying stent implantation and healing [11, 12]. Twenty-one male New Zealand White White Rabbits (2kg, NZW, Charles River Laboratories) were maintained in individual confinements with ample food and water supply. Animal health was monitored daily and animals were allowed regular activity periods outside of the cage. Local and systemic analgesics were employed liberally to limit any suffering and distress. Humane endpoints were continuously monitored by the animal care staff and veterinarians. Rabbits were euthanized by lethal doses of pentobarbital sodium. No mortality occurred outside the planned euthanasia or humane endpoints. Rabbits were randomly assigned by online randomization tool to undergo implantation in the abdominal aorta of either a bioresorbable vascular scaffold (BVS) (n = 11) or drug-eluting stent (DES) (n = 10). A convenience sample was employed based on available animals. Stent implantations were performed during daytime hours in a dedicated operating suite with induction by propofol and subsequent general inhaled anesthesia with vascular access achieved via femoral cut-down approach enabling placement of a 6-French femoral access sheath with subsequent closure by femoral ligation as previously reported [13, 14]. Intravenous unfractionated heparin (150U/kg) is administered intra-procedurally to prevent thrombosis, while post-stent implantation rabbits were maintained on dual anti-platelet therapy with rectal acetylsalicylic acid (ASA) 10mg/kg daily and intra-dermal clopidogrel 14mg daily to prevent stent thrombosis. OCT analyses were then performed using saline and contrast as flush solutions at two time-points–(i) immediately following stent implantation and (ii) 6-weeks post-implantation at the time of sacrifice. Three randomly selected unmatched images deemed representative of their respective scans were flagged for qualitative and quantitative evaluation in both the saline and contrast groups.

### *In vivo* qualitative analysis

Identical de-identified images in both the saline and contrast groups underwent subjective grading to compare diagnostic quality and the presence of artifacts between the two groups. Scoring was completed by blinded, independent, and trained evaluators based on five pre-determined criteria in a binary fashion (no = 0, yes = 1), in keeping with similar scoring systems [15]. Cumulative quality and artifact scores were assigned to each image ranging from 0 to 4. The following criteria were used to determine diagnostic quality: (1) the presence of a clear border between lumen and luminal wall; (2) the presence of a defined border between the tunica interna and tunica media; (3) the ability to confidently identify whether stent struts were covered or uncovered; and (4) whether the overall image quality was satisfactory for diagnostic interpretation. To assess for difference in the observed artifacts between saline and contrast, four pre-established criteria were employed. Cumulative artifact scores ranging from 0 to 4 were calculated from the binary scores based on the presence or absence of the following: (1) blood swirl or speckle occupying >50% of the lumen, or the presence of thrombus; (2) sew-up artifact; (3) ghost reflections on >1 strut; and (4) saturation artifact.

### *In vivo* quantitative analysis

Dimensional analysis similar to the bench top model was performed to assess both the luminal area and diameter with saline and contrast *in vivo*. Area and diameter were calculated via the automatic area function or manually if the software was unable to detect the luminal wall. The number of stent struts and coverage of struts were manually counted based on the presence of strut and strut reflection shadow. All measurements were completed by independent reviewers blinded to the treatment groups.

### Statistical analysis

Continuous variables are reported as either mean (±SD) or median (IQR). Categorical variables are reported as either frequencies and/or percentages. All plotted relationships were linear with appropriate linear trendline fitting applied and the resulting line of best fit and R^2^ displayed for each. The linear equation derived from the line of best fit was then used to derive correction formulas for each contrast concentration with validation performed in a separate cohort. Categorical variables were compared using chi-square tests, whereas continuous variables were compared using either Student t-tests or Mann-Whitney *U* tests, where appropriate.

## Results

### Bench top dimensional analysis

**Fig 2:**
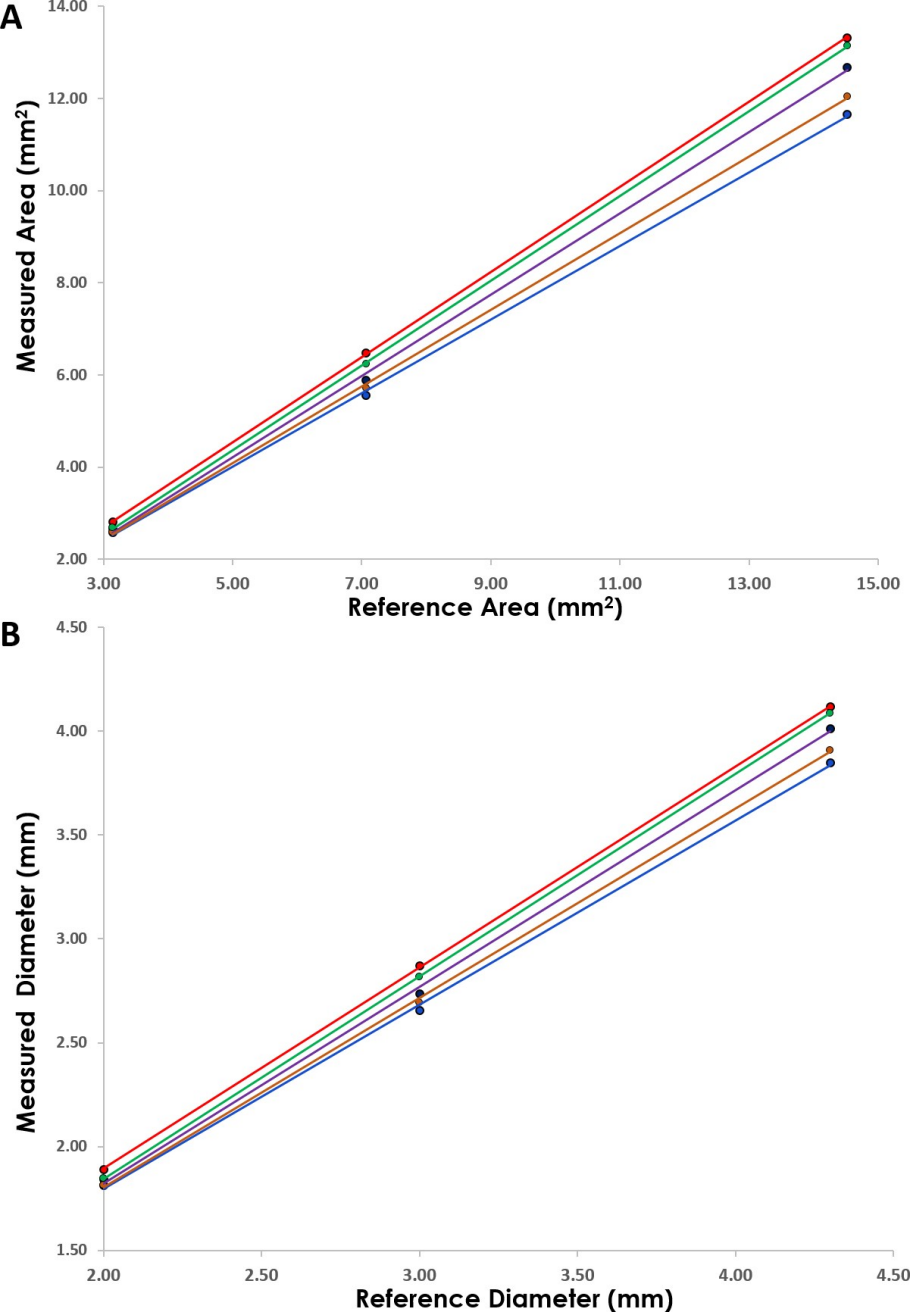
Measured versus reference dimensional analysis. Dimensional analysis of measured areas (A) and diameters (B) via OCT compared to reference nylon tubing dimensions. Demonstrates linear relationship with differing slopes varying by the different flush solutions employed for each. Refer to Table 1A and 1B for trendline formulas and correlation coefficients. Red– 100% contrast, Green– 75% contrast, Purple– 50% contrast, Orange– 25% contrast, Blue– 0% contrast (pure saline).

We first set out to empirically derive adjustment coefficients for dilutions of contrast on the bench top. Nylon tubing was assessed with varying concentrations of saline/contrast and the generated areas and diameters were plotted as a function of the known reference area and diameters of the tubing, demonstrating a linear relationship across all dilutions (Fig 2). As well, a progressively decreasing slope was noted as one reduced contrast content for both dimensions (Fig 2; Table 1A and 1B). The calculated linear relationships of the measured versus reference dimensions (Table 1A and 1B) were then used to derive a conversion formula for the reported contrast dilution ratios. These conversion formulas allow for the calculation of the corresponding 100% contrast dimension (Table 2A) or the reference dimension (Table 2B) for any measured dimension at a pre-defined contrast percentage (defined as X in Table 2A and 2B). We then applied these correction formulas to the raw data obtained in a separate validation cohort by plotting the corrected values versus the corresponding 100% contrast and reference values for both area and diameter. With this approach, a 1:1 relation of corrected dimension to contrast or reference dimension would indicate a reliable correction. Indeed, this was observed with validation slopes ranging from 0.98–1.03 with R^2^ all greater than 0.97 (Table 2A and 2B), indicating a reliable adjustment for area and diameter across the varying flush solution compositions.

**Table 1:** Dimensional analysis (A) measured versus reference area (B) measured versus reference diameter.

**A**	**Reference (mm**^**2**^**)**	**3.14**		**7.07**		**14.52**		**Measured versus Reference**	
**Measured (mm**^**2**^**)**	**Contrast (%)**	**Mean (mm**^**2**^**)**	**SD**	**Mean (mm**^**2**^**)**	**SD**	**Mean (mm**^**2**^**)**	**SD**	**Relation**	**R**^**2**^
	**100**	2.80	0.07	6.48	0.21	13.32	0.71	y = 0.9235x - 0.077	1
	**75**	2.68	0.11	6.24	0.22	13.15	1.01	y = 0.9202x - 0.2303	1
	**50**	2.67	0.09	5.89	0.31	12.67	0.60	y = 0.8829x - 0.203	0.9993
	**25**	2.59	0.08	5.71	0.41	12.05	1.05	y = 0.8331x - 0.0844	0.9997
	**0**	2.59	0.12	5.55	0.29	11.66	0.80	y = 0.7997x + 0.0051	0.9996
**B**	**Reference (mm)**	**2.00**		**3.00**		**4.30**		**Measured versus Reference**	
**Measured (mm)**	**Contrast (%)**	**Mean (mm)**	**SD**	**Mean (mm)**	**SD**	**Mean (mm)**	**SD**	**Relation**	**R**^**2**^
	**100**	1.89	0.02	2.87	0.05	4.12	0.11	y = 0.968x - 0.0426	0.9999
	**75**	1.85	0.04	2.82	0.05	4.09	0.16	y = 0.9742x - 0.1036	1
	**50**	1.84	0.03	2.73	0.07	4.01	0.10	y = 0.946x - 0.0696	0.9993
	**25**	1.82	0.03	2.69	0.10	3.91	0.18	y = 0.9112x - 0.0194	0.9997
	**0**	1.81	0.04	2.66	0.07	3.85	0.13	y = 0.8859x + 0.0254	0.9995

**Table 2:** Conversion factors (A) Adjust measured sizing to contrast sizing (B) Adjust measured sizing to reference sizing.

Contrast (%)	Derivation (N = 45)	Validation (N = 45)	
**A**			
**Area**	**Conversion formula**	**Slope**	**R**^**2**^
**75**	Contrast = ((0.9194(X+0.1584))/0.9063)-0.0391	1.0228	0.9952
**50**	Contrast = ((0.9194(X+0.1149))/0.8706)-0.0391	1.0169	0.9966
**25**	Contrast = ((0.9194(X+0.0928))/0.831)-0.0391	1.0029	0.992
**0**	Contrast = ((0.9194(X-0.0501))/0.7926)-0.0391	1.0127	0.996
**Diameter**	**Conversion formula**	**Slope**	**R**^**2**^
**75**	Contrast = ((0.9708(X+0.1340))/0.9862)-0.0518	0.9824	0.996
**50**	Contrast = ((0.9708(X+0.1060))/0.9577)-0.0518	0.9796	0.9961
**25**	Contrast = ((0.9708(X+0.0255))/0.9147)-0.0518	1.0003	0.995
**0**	Contrast = ((0.9708(X-0.0057))/0.8928)-0.0518	0.9902	0.996
**B**			
**Area**	**Conversion formula**	**Slope**	**R**^**2**^
**100**	Reference = (X+0.0391)/0.9194	1.0094	0.9905
**75**	Reference = (X+0.1584)/0.9063	1.0313	0.9834
**50**	Reference = (X+0.1149)/0.8706	1.0287	0.9913
**25**	Reference = (X+0.0928)/0.831	1.0055	0.9692
**0**	Reference = (X-0.0501)/0.7926	1.0184	0.9791
**Diameter**	**Conversion formula**	**Slope**	**R**^**2**^
**100**	Reference = (X+0.0518)/0.9708	0.9942	0.9941
**75**	Reference = (X+0.1340)/0.9862	0.9757	0.9881
**50**	Reference = (X+0.1060)/0.9577	0.9756	0.9936
**25**	Reference = (X+0.0255)/0.9147	0.9924	0.9847
**0**	Reference = (X-0.0057)/0.8928	0.9845	0.9904

### *In vivo* quantitative analysis

**Fig 3:**
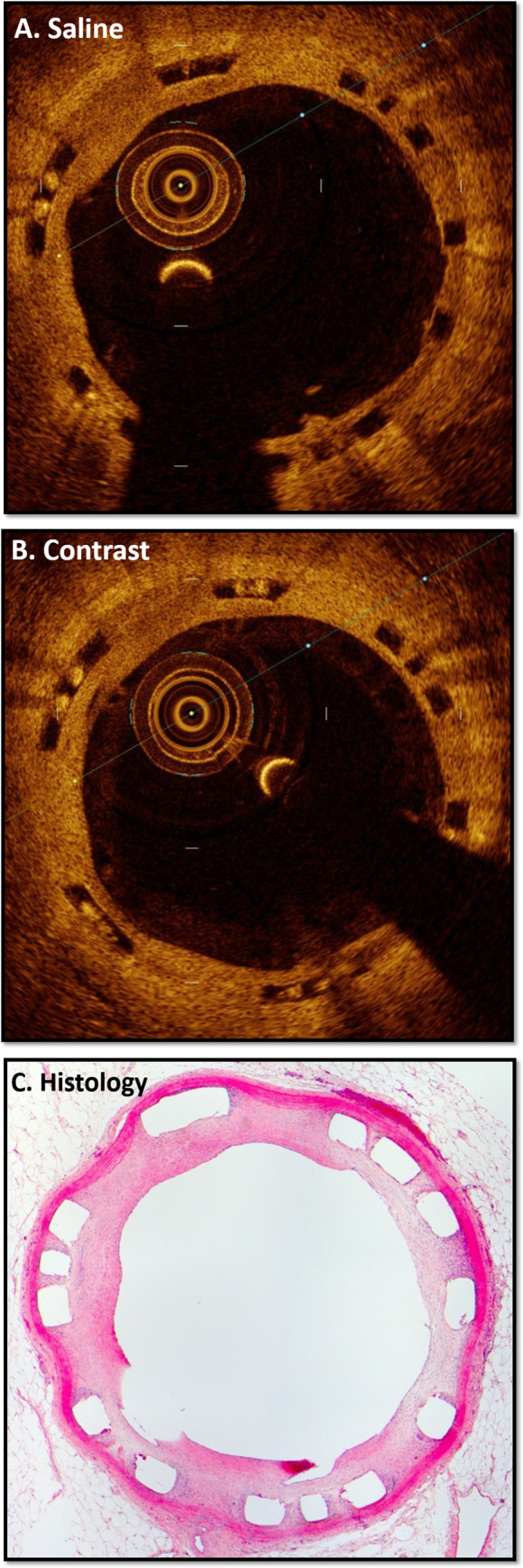
*In vivo* comparison of OCT versus traditional histology. Images of identical rabbit arterial segment with scaffold in situ and subsequent arterial healing and neointima formation. Specifically note of robust virtual histology with OCT utilizing both 100% saline (A) and 100% contrast (B) flushing agents when compared to traditional histology with haematoxylin and eosin (H&E) staining (C).

**Fig 4:**
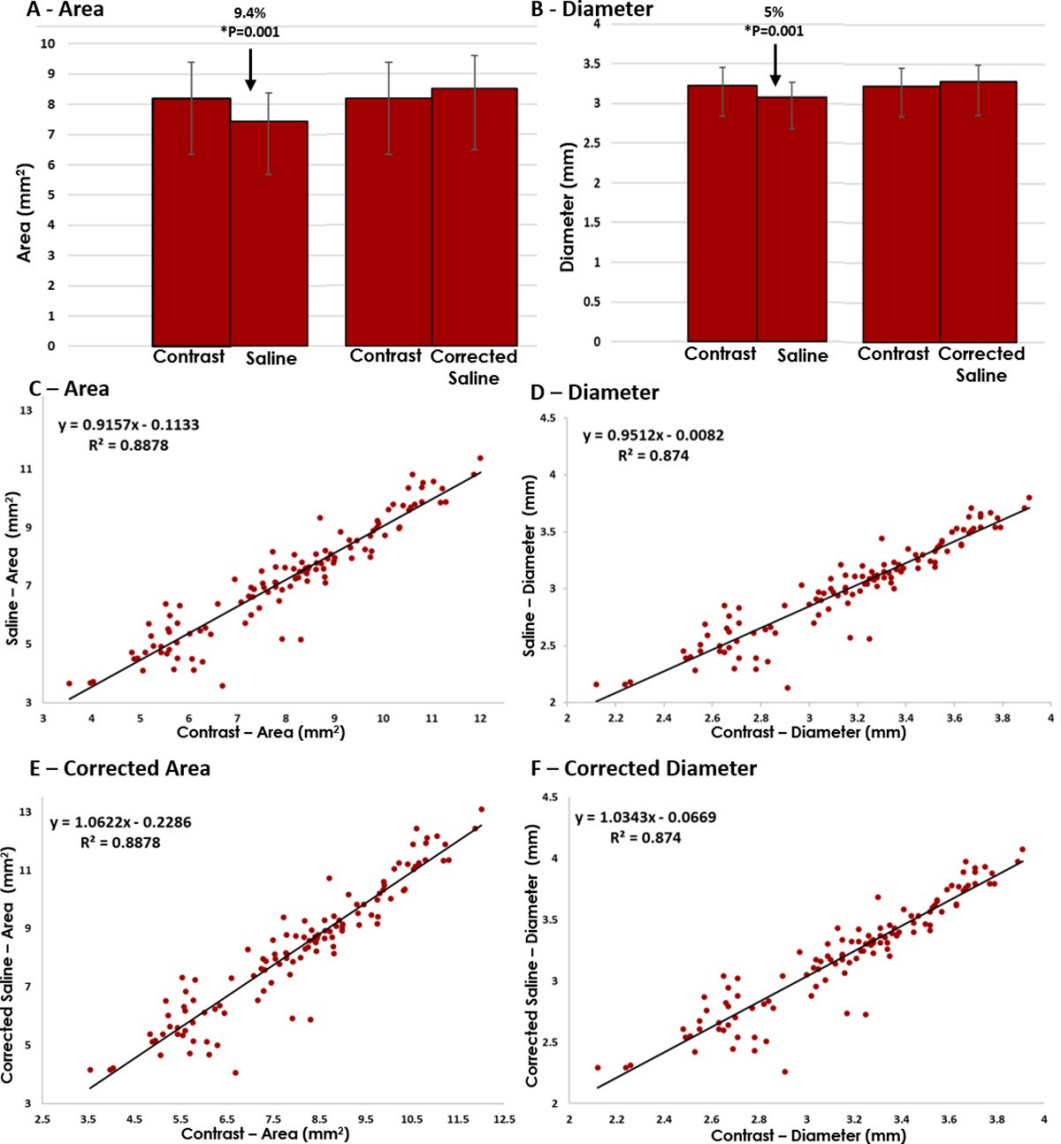
*In vivo* quantitative dimensional analysis. Dimensional analysis of area (A) and diameter (B) in rabbit model of intravascular OCT. Note of a significant 9.4% reduced area and 5% reduced diameter in the saline versus contrast cohorts. Following application of empirically derived correction factors no further significant difference between either cohorts. Graphical depiction of individual data points for saline versus contrast assessments for both area (C) and diameter (D) demonstrating linear relationship with slopes of 0.9157 and 0.9512 respectively. Following application of empiric correction factors note of augmented slope to 1.0622 and 1.0343 respectively for area (E) and diameter (F).

Following our bench top assessment, we sought to assess the impact of imaging media in an *in vivo* model. This model utilizes stent implantation in the abdominal aortas of New Zealand White Rabbits with intravascular OCT assessment capable of generating histological grade imaging with both 100% saline and 100% contrast imaging media (Fig 3). Findings in this model mirrored our bench top findings with the saline cohort demonstrating a 9.4% reduction in area [7.43 (5.67–8.36) versus 8.2 (6.34–9.39 mm^2^), p = 0.001] and 5% reduction in diameter [3.08 (2.68–3.26) versus 3.23 (2.84–3.46) mm, p = 0.001] in comparison to its contrast counterpart (Fig 4A and 4B).

Next, we applied the bench top derived and validated dimensional correction formulas (Table 2A) to the data generated from our *in vivo* model. Specifically, we applied the correction formula for 100% saline to 100% contrast [Contrast = ((0.9194(X-0.0501))/0.7926)-0.0391] to the raw data obtained *in vivo* with 100% saline, generating corrected saline values. This correction eliminated differences between corrected saline and contrast in both area [8.52 (6.48–9.60) vs 8.20 (6.34–9.39) mm^2^, 9.4% to 3.9% absolute difference, p = 0.35, Fig 4A) and diameter [3.29 (2.86–3.49) vs 3.23 (2.84–3.46) mm, 5% to 1.9% absolute difference, p = 0.40, Fig 4B). Similarly, when the corrected saline values were plotted as a function of the measured contrast values, they demonstrated a strong 1:1 linear relationship indicating robust correction for both area (slope = 1.06, R^2^ = 0.89, Fig 4E) and diameter (slope = 1.03, R^2^ = 0.87, Fig 4F) when compared to uncorrected dimensions with saline versus contrast (Fig 4C and 4D).

Last, we used the *in vivo* images to quantify stent struts and arterial healing post stent implantation. The ability to identify stent struts and describe their appearance (i.e. exposed or covered) is of particular importance to assessing stent deployment and pathology. We did not observe any differences between saline or contrast with regards to the total number of stent struts, exposed struts, or covered struts (Table 3). Furthermore, neointima quantification by OCT imaging with saline and contrast similarly demonstrated no differences between groups (32.3+/-6.46 versus 30.3+/-5.56%, p = 0.25) (Table 3).

**Table 3:** *In vivo* quantitative stent analysis.

	Saline		Contrast		
	Mean	SD	Mean	SD	p
**Strut Assessment (N)**	117		117		
Exposed struts	5.81	6.63	5.44	6.06	0.92
Covered struts	6.29	6.78	6.79	6.74	0.55
Total strut number	12.10	4.42	12.23	3.68	0.9
**Neointima assessment (N)**	25		25		
Neointima size (%)	32.3	6.46	30.3	5.56	0.25

### *In vivo* qualitative analysis

**Fig 5:**
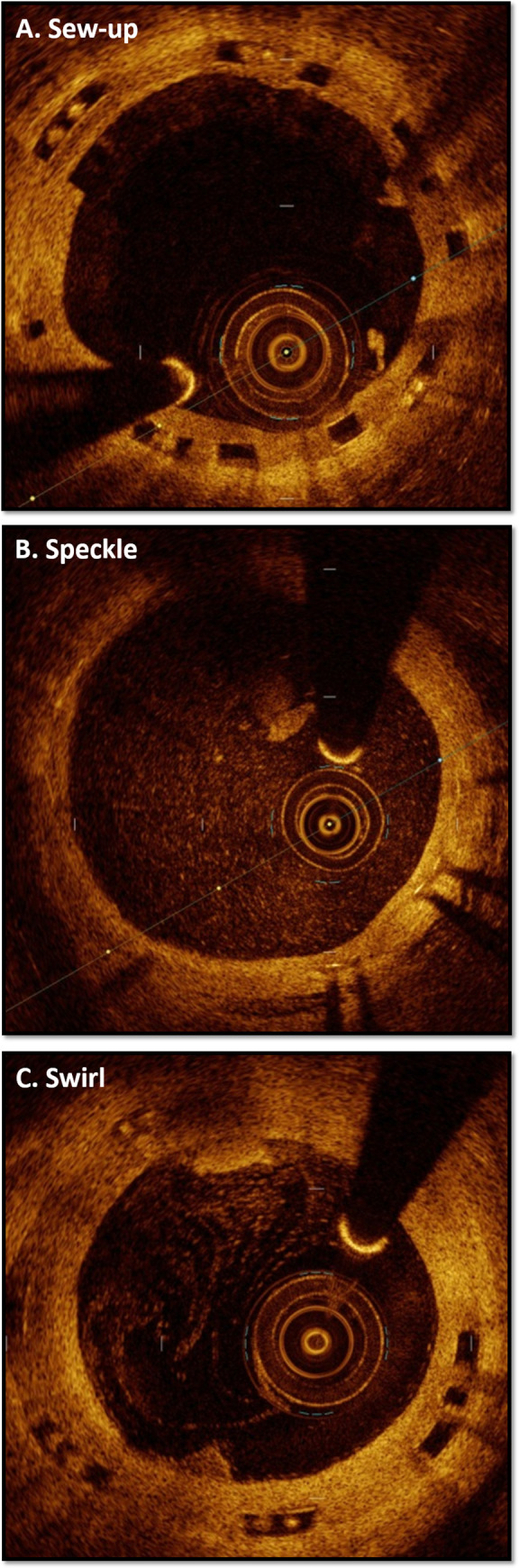
Artefacts with intravascular OCT. Examples of common artefacts with intravascular OCT including sew-up artefact (A) related to motion, as well as speckle (B) and swirl (C) artefacts related to insufficient intravascular blood flushing.

Based on the 4-point quality scoring system, all *in vivo* images for saline (n = 117) and contrast (n = 117) were assessed by independent reviewers for each quality component to generate a summative overall quality score (Table 4). Ultimately, no significant differences between any of the individual quality components, or in the overall quality scores were observed. Similarly, independent reviewers evaluated both the saline (n = 117) and contrast (n = 117) cohorts for the presence of common artefacts known to compromise intravascular OCT imaging (Table 5; examples are demonstrated in Fig 5). Overall, no significant differences in any of the artefacts screened for were demonstrated between either groups. Both saline and contrast yielded image quality on par with histological-grade images, with corresponding histological sections demonstrating striking similarities (Fig 3).

**Table 4:** *In vivo* qualitative analysis–quality score.

	Saline	%/SD	Contrast	%/SD	p
N	117		117		
**Luminal border**	109	93.1	108	92.3	1.00
**Intima/media border**	43	36.8	37	31.6	0.49
**Stent strut**	100	85.4	95	81.2	0.48
**Diagnostic quality**	99	84.6	93	79.5	0.39
**Overall score**	3.0	1	2.85	1.06	0.06

**Table 5:** *In vivo* qualitative analysis–artefact score.

	Saline	%	Contrast	%	p
Number	117		117		
**Blood score**	23	19.7	12	10.3	0.067
**Sew-up**	0	0.0	1	0.9	1.000
**Ghost-strut**	0	0.0	0	0.0	-
**Saturation**	0	0.0	0	0.0	-

## Discussion

Intravascular OCT provides histological-grade intracoronary assessment, but requires clearance of intraluminal blood via flushing with viscous contrast solution, which carries risks [16]. Preliminary work has investigated the utility of varying flush solutions including dextran and saline with promising results [17, 18]. However, a thorough preclinical assessment of the qualitative and quantitative properties has yet to be reported. Herein, we provide empirically-derived and validated correction formulas for dimensional analysis for saline-based imaging. Moreover, we demonstrate the utility of these correction formulas in an *in vivo* setting for dimensional analysis while also demonstrating no significant impact on quantitative and qualitative stent assessments in a preclinical rabbit model.

### Dimensional analysis

Our bench top and *in vivo* models enable assessment of the impact of the contrast-content of imaging media on measured intravascular areas and diameters. Both models demonstrate a significant difference with respect to the dimensions reported between saline and contrast in keeping with previous reports [19]. The observed differences in dimensions are likely related to the varying refractive indices between saline (1.33) and contrast (1.44), with most commercial systems employing a refractive index of 1.4 for dimensional calculations [19]. In our bench top model we similarly noted a 10% difference in sizing that, once corrected, yielded no difference between the two groups (4%). Similarly, when assessed in the *in vivo* setting no differences in dimensions remained following correction, with only a 4% difference in area and 2% difference in diameter remaining. This is in keeping with previous work in a swine model that reported a variance in area of 18% that was reduced to 2.9% with correction based upon the refractive index of the solution [19]. Our empirically-derived correction factors thus enable robust adjustment for a broad range of contrast dilutions across a physiologically-relevant range of luminal diameters.

### Qualitative and quantitative assessments in diagnostic quality

In addition to vessel sizing, OCT is commonly performed to clarify unclear anatomy, assess for dissections, and thrombus, and/or plaque morphology. Thus, acquiring a diagnostic image to permit assessment of the vessel architecture is paramount for imaging performance. Accordingly, we assessed qualitative measures in an established rabbit stenting model by performing a standardized assessment of a reviewers’ ability to identify key vessel architectural features and artefact presence [15]. In our study, we found no significant differences between saline and contrast in any of the individual quality components, nor in the overall sum quality score suggesting that saline performs comparably to contrast in our system. Similarly, reviewers assessed for the presence of artefacts and did not identify any differences between saline and contrast, though there may be a suggestion of more blood artefact in the saline cohort. This is most likely related to reduced flushing efficacy with saline owing to its >10-fold lower viscosity than iodinated contrast [19, 20]. Indeed, the dynamics of flushing has been explored previously with viscosity, flow rate and flush duration identified as the predominant factors impacting blood displacement [19]. We employed a standardized flushing protocol with a rate sufficient to clear blood and to initiate auto-triggering of the OCT run. This rate was then maintained for a fixed duration for each run; therefore, any suggested any variance in image quality would be most likely related to differing viscosities of the two agents. To overcome the reduced viscosity, one could improve blood clearance by either increasing the flush duration and/or flow rate [19]. Moreover, this effect is likely magnified in the rabbit model as flushing is performed in a retrograde fashion against aortic blood flow as opposed to an antegrade approach in human coronaries.

### Study limitations

Our work is not without limitations. First, our bench top model is based on standardized internal diameters, which, while stringently manufactured and controlled for, still report variances in sizing of ±0.08mm/0.003”. However, this variance should minimally impact our adjustment coefficients, given its low magnitude and equal distribution in both groups. Second, the importance of arterial blood clearance is paramount for robust image production. The abdominal aorta of rabbits is an established model for assessment of stent implantation and healing [11, 12]. However, while it provides a comparable physiologic system and dimensions to human coronary arteries it is less complex than the human coronary tree. Clinical performance in human coronaries would be needed to corroborate our findings and to optimize and validate saline as a dedicated contrast agent for OCT imaging during diagnostic and interventional coronary procedures. While anecdotal use of saline is reported, varying parameters such as volumes, injection parameters and coronary complexity would need to be standardized for evaluation.

## Conclusions

In summary, saline generates reduced dimensions in a linear fashion, enabling robust correction to contrast values. Our model suggests no significant difference in image quality with saline flushing on account of its reduced viscosity, while any differences could likely be mitigated by varying the flush rate and/or duration. Clinical studies investigating the efficacy of saline as a contrast agent for OCT imaging are warranted.

## Supporting information

S1 Dataset (XLSX)
